# A Heuristic to Create Prosumer Community Groups in the Social Internet of Energy

**DOI:** 10.3390/s20133704

**Published:** 2020-07-02

**Authors:** Víctor Caballero, David Vernet, Agustín Zaballos

**Affiliations:** Engineering Department, Universitat Ramon Llull (URL), La Salle, 08022 Barcelona, Spain; david.vernet@salle.url.edu (D.V.); agustin.zaballos@salle.url.edu (A.Z.)

**Keywords:** social internet of things, social internet of energy, smart grid, clustering, prosumer community groups

## Abstract

Contrary to the rapid evolution experienced in the last decade of Information and Communication Technologies and particularly the Internet of Things, electric power distribution systems have remained exceptionally steady for a long time. Energy users are no longer passive actors; the prosumer is expected to be the primary agent in the Future Grid. Demand Side Management refers to the management of energy production and consumption at the demand side, and there seems to be an increasing concern about the scalability of Demand Side Management services. The creation of prosumer communities leveraging the Smart Grid to improve energy production and consumption patterns has been proposed in the literature, and several works concerned with scalability of Demand Side Management services group prosumers to improve Demand Side Management services scalability. In our previous work, we coin the term Social Internet of Energy to refer to the integration between devices, prosumers and groups of prosumers via social relationships. In this work, we develop an algorithm to coordinate the different clusters we create using the clustering method by load profile compatibility (instead of similarity). Our objective is to explore the possibilities of the cluster-by-compatibility heuristic we proposed in our previous work. We perform experiments using synthetic and real datasets. Results show that we can obtain a global reduction in Peak-to-Average Ratio with datasets containing up to 200 rosumers and creating up to 6 Prosumer Community Groups, and imply that those Prosumer Community Groups can perform load rescheduling semi-autonomously and in parallel with each other.

## 1. Introduction

Global energy demand is expected to increase by 50% from 2018 to 2050 [1]. Nevertheless, electric power distribution infrastructures have remained unchanged for a long time, in opposition to the fast evolution of Information and Communication Technologies (ICTs) during the last decade, such as the Internet of Things (IoT) [2]. Traditional and non-renewable energy sources supply most global energy demand; however, non-renewable energy sources are starting to be insufficient and produce harmful climate changes in our ecosystem [3]. Therefore, society is increasing the adoption of renewable energy sources [4]. The Smart Grid (SG) refers to the addition of ICT infrastructure to the electrical domain (and the traditional electricity grid), and enables new services and opportunities that are guiding a revolution in the energy field [5,6,7,8].

Motivated by the convergence of the electrical grid, ICTs, and the possibility to generate individual energy through Renewable Energy Source (RES), new actors emerge on the SG. According to [9], prosumers are “those customers who decide to invest in distributed energy resources (mostly solar PV) for a variety of reasons and […] can satisfy a portion of their electricity demand and […] produce more than they consume, […]. Apart from helping to match supply and demand, sources of flexibility can also assist in various ancillary services such as frequency and voltage profile control […]”.

Flexible sources of energy enable management services at the demand side; those services aim at optimizing the use of energy resources. Thus, Demand Side Management (DSM) refers to the management of energy consumption and production at the demand side; for instance, residential energy users might modify their consumption behavior to reduce greenhouse gas emissions. Therefore, a new type of active actor, the prosumer (which produces and consumes energy), is expected to be the primary agent in the Future Grid—rather than the utility. Consequently, the number of nodes that need to coordinate (or be coordinated) to reduce greenhouse gas emissions increases.

Furthermore, prosumers are not the last nodes that need coordination, but the energy-consuming devices and appliances they own. There seems to be an increasing concern about the scalability of DSM services [10,11]. To tackle such challenge, we envision a synergy between SIoT [12] and SGs, and, in our previous work [8], we coined the term Social Internet of Energy (SIoE) to refer to this union. Instead of focusing on concrete DSM algorithms, our focus is on characterizing a social network of prosumers and their smart devices and appliances.

Works such as [13,14] consider “prosumer communities [that] aim to transform traditional consumers to become active prosumers thereby improving the efficiency of the smart grid and offering economic, operational and environmental benefits.” [13]. Additionally, a distributedClean Energy Community (CEC) is “a network of households and businesses that generate or own distributed generation individually, connected through a controlling entity either physically or virtually, and sharing the same rules in supplying and consuming electricity within the network” [14]. Both definitions are not exclusive, and indeed [13] proposes the first definition to encompass similar related terms.

Our vision is to enable an overlay social network of smart devices that facilitates communication and discovery between devices, prosumers and Prosumer Community Groups (PCGs). Prosumers in a PCG might be from different geographical locations (and bounded by, for example, the operational region of the utility); however, they should share a common goal such as optimal energy management [13]. Also, in the presence of behavioral changes on prosumer’s energy consumption, the social network of prosumers we envision could acquire a new configuration to facilitate energy management at the demand side.

One of the last and novel configurations of IoT frameworks could be able to provide the overlay social network of smart devices. According to a generational analysis of the IoT [2], the SIoT [12,15] belongs to the third and most recent generation of IoT frameworks. The SIoT envisages interactions between humans and machines, in addition to human-to-human and machine-to-machine interactions; the former enabled by Social Network Services (SNSs), and the latter approached by the IoT [8]. In the future SIoT, nodes of the same network represent humans and devices that provide services [16]—they are not logically separated.

On [8], we described the kind of SIoT relationships devices and owners need to establish to create a network of prosumers and PCGs. To group prosumers, we need a heuristic based on the attributes of prosumers, one of these attributes is their energy-consumption behavior. Because one of the DSM services goals is to reduce the energy peaks that occur when prosumers consume energy at the same time, we aimed to group them by their energy profile compatibility, instead of grouping them by energy profile similarity.

The heuristic pursued two goals at the same time: first, to reduce peaks locally; and, secondly, to group prosumers that have opposite energy-consumption behaviors—by their compatibility. The rationale to group by compatibility is as follows. Ideally, if two prosumers consume and produce the same amount of energy at exactly opposite moments in a timeline, the aggregate load curve they generate is zero during all the timeline (because consumption and production of each prosumer negate each other). Moreover, if they are instructed to make any change on the time of use of their appliances, the change would be smaller than if they were grouped by similarity.

Therefore, in this work, we develop an algorithm to coordinate the different clusters we create using the clustering method by prosumer compatibility (which we describe in [8], and summarize in Section 4.2). We consider a simple load model, similar to the one the authors in [17,18] use that includes fixed and time-shiftable loads. Our main objective is to explore the possibilities of the cluster-by-compatibility heuristic we propose. Then, in future work, we will try to find answers as to why some cluster configurations may achieve a lower Peak-to-Average Ratio (PAR) than other configurations. For the first time, and to the best of our knowledge, the results show that we can achieve an optimal cluster configuration using the clustering-by-compatibility heuristic.

This article is organized as follows. Section 2 sets a background on SGs, PCGs, and DSM. Section 3 describes works related to scalability of DSM services in the SG and complements the positioning of the article we give in Section 1. Section 4 describes the load, clustering and rescheduling model we use. We perform experiments and analyze the results in regard to our DSM model and heuristic on Section 5. Finally, we summarize our conclusions in Section 6.

## 2. Background

### 2.1. Future Grid and New Era Technologies

Contrary to the rapid evolution experienced in the last decade of ICTs and particularly the IoT [2], electric power distribution systems have remained exceptionally steady for a long time. Nevertheless, energy demand in the world is increasing rapidly. Worldwide energy consumption is expected to increase by nearly 50% from 2018 to 2050 [1]. Most of the energy demand is met by non-renewable energy resources which are starting to be insufficient and produce undesirable climate changes that harm the world we live in [3].

Indeed, the report of the United Nations on Sustainable Development Goals progress [4] states: “[…] since 2012 […] the growth of renewables (has) outpaced the growth of total energy consumption”. We can find further evidence in relation to the COVID-19 situation: while there was a decrease of 3.8% in energy demand during the first quarter of 2020 due to lockdown, “renewables were the only [energy] source that posted a growth in demand, driven by larger installed capacity and priority dispatch” [19]. Hence, society is moving towards the use of renewable and sustainable energy sources. Consequently, the increase in energy demand will not be manageable unless the traditional electricity grid evolves.

The convergence of the ICT infrastructure to the electrical domain (the SG), has enabled new services and opportunities (e.g., customer-side generation, real-time energy consumption, and accurate grid monitoring), which are guiding a significant revolution in the energy field [5,6,7,8]. The SG envisions the improvement traditional of electric infrastructures in several dimensions [20] such as information, scalability and business models, to meet the highest standards of power quality and ensure that the electric power grid is cost-effective and sustainable [21].

The SG has led to the rise of new agents and components. The new energy user does not only consume energy but performs a crucial role in the SG. Prosumer (energy producers and energy consumers [13]) are easily the most important creators of value within the SG, as they are the last link in the electricity value chain. Prosumers are active energy users in the Future Grid. The most important connections for prosumers in the electricity value chain are the dso or the aggregator/retailer in addition to Energy Services Companies (ESCOs), Virtual Power Plants (VPPs) [22], Microgrids (MGs) [22] or PCGs [13,23], which are also new components in the energy market value chain [24]. Those components are key in managing the increase of energy demand and meet the demand with energy gathered from RESs. They range from energy-oriented commercial businesses, ESCOs; to systems that operate Distributed Energy Resources (DERs), VPP; that can even operate autonomously as a single unit detached from the main grid, MGs; to PCGs, that describe prosumer collaboration towards a common goal.

Those new agents and components of the SG could become the most enriched elements thanks to their integration with ICTs, that upgrade information and communication capabilities [7]. However, the upgraded features at the end-user side come at the cost of requiring more technology. Due to the complexity and magnitude (i.e., stringent levels of service reliability and availability in large-scale areas), and new agent profiles in the SGs, practitioners have recently confronted the digital transformation of electric networks by proposing flexible and future Internet-based architectures [21].

The SG has a heterogeneous nature which demands system architects to consider multiple telecommunication technologies. sg are deployed in hostile wireless communication environments [25]. Aware protocols (cognitive radio techniques) [26] are needed to reduce communication delay [25]; meet qos needs in terms of bandwidth, data reliability, and delay; improve energy harvesting techniques [26]; and provide reliable sensing in a distributed environment [27]; to minimize interoperability issues between heterogeneous communication networks [7,28].

The SG is considered a special use case of the IoT, with its own requirements, coined as the Internet of Energy (IoE) [29]. A common challenge for SG and IoT is the accessibility of deployed sensors and actuators. As long as those devices are connected to the Internet and included in the IoT, they are potentially accessible. Nonetheless, these heterogeneous devices expose diverse interfaces, which renders access to those resources difficult and non-scalable.

As stated, one of the most relevant agents in the SG and Future Grid are prosumers, individual users that consume and produce energy. A prosumer is “an energy user who generates renewable energy in his/her domestic environment and either store the surplus energy for future use or trades to interested energy customers in smart grid” [13,30]. To meet worldwide energy demand using RESs, recent literature proposes to group energy users (prosumers) to optimize services and opportunities, such as energy generation, demand-side or storage resources and to increase individual der visibility; while allowing these groups perform those services autonomously. Those prosumer communities are often called pcg, and they pursue a mutual goal and compete in the energy market as a group. Other related terms, such as Electricity Prosumer Communities (EPCs) [31], Integrated Community Energy Systems (ICESs) [32] and CECs [14] are used to refer to the same concept [13].

Prosumers, rather than the utility, are expected to be the primary agents in the Future Grid. Management services at the demand side (prosumers’ side) aim at optimizing the use of energy resources. Thus, DSM refers to the management of energy consumption and production at the demand side. There are several DSM services that usually involve an agreement with the utility and prosumers. Those services range from using price signals to control or bias the consumption pattern of prosumers (Real Time Pricing (RTP) [33]), to allowing the utility to remotely control the operation and consumption of certain appliances (Direct Load Control (DLC) [33]). Energy usage optimization pursues the reduction of energy demand peaks to reduce blackouts and successfully manage der such as storage systems (batteries, Electric Vehicle (EV)) and energy gathered from RES. Energy from RES cannot be gathered on demand since it depends on climate factors. Therefore, energy storage systems must store surplus energy from RES to provision the grid on demand. Effectively managing consumption patterns leads to a reduction in energy demand peaks and a decrease in overall energy consumption.

### 2.2. New Era Technologies

The IoT [2,34] is emerging as the new era technology, where before were the Internet and the Web. The IoT is one of the consequences of the Internet—that which allowed to create an intercommunicated world and the World Wide Web that builds on top of a World Wide Network. As science and technology advancements allowed, more and more devices were being connected to the World Wide Network, a hyper-connected world was and is still emerging. The hyper-connected world was coined under the umbrella term IoT, or for more concrete use cases, Internet of X. Where X receives the value of vehicles, people, radios or energy, among other names.

Nevertheless, more often than not, these field-specific IoT deployments operate in silos. A lack of homogenization of data and protocols used by smart devices prevents local IoT deployments to inter-operate and pose a challenge to realize the full potential of the IoT. “Interoperability is considered to be the ability of two or more systems or components to exchange information and data, and use the exchanged information and data without special effort by either system, or without any special manipulation.” [35]. The Web and its technologies serve humankind well in facilitating the access and globalization of information. It is envisioned by some authors as the homogenization technologies through which rendering the IoT easily and homogeneously accessible. Yet, and although still in development these days, the Web of Things (WoT) [36], as it is called, is far from one solution fits all.

The WoT answers the challenge of protocol and data homogenization, which enables accessibility, findability, shareability and composition of WoT [36], enhancing the scalability of the IoT. A system is space-time scalable “if it continues to function gracefully as the number of objects it encompasses increases by orders of magnitude. A system may be space-time scalable if the data structures and algorithms used to implement it are conducive to smooth and speedy operation whether the system is of moderate size or large.” [37]. The wot enables data accessibility and findability using already available Web technologies. However, the discovery of billions of objects is challenging. Tackling scalability and diversity (heterogeneity) is not an easy task. wot search engines should support local-scale and global-scale search at the same time while also considering diversity of search queries and services. For that matter, wot search engines seem to be moving towards a distributed approach  [38].

Recently, another approach to scalable discovery has gained momentum. The approach is based on the small world phenomenon [39], which states that people is connected by short chains of acquaintances and thus, creating a worldwide network of connections where there is always a short path between the source node and the target node. After all, if there is a natural phenomenon of such characteristics that allows scalability and findability, why cannot devices create the same kind of network? The term SIoT [12,15] refers to just that, a social network of things; similar to the social structure that emerged from the ones we have created for humans, namely Facebook, Google Plus or Twitter, but for connected devices.

The IoT enables smart objects or *things* to connect to the Internet, enabling them to be accessed as their heterogeneity allows. This layer is heterogeneous in that smart devices are built by different vendors and use different formats to structure their data and different protocols to communicate. It is also the main reason for the existence of IoT silos; specific IoT deployments, while interconnected locally for specific use cases, are not able to interact with the external world directly due to their heterogeneity—although they are connected to the Internet. The WoT and the SIoT build on top the IoT infrastructure layer. These two frameworks are not on top of the other and instead they provide complementary mechanisms to overcome the challenges of IoT.

The WoT provides homogenized access to smart devices through the semantic Web [40,41,42] and Web protocols such as Hyper-Text Transfer Protocol/Secure (HTTP/S) [7,42]. Homogeneous access at the semantic level allows devices to be found as they share a common framework that describes them. The accessibility layer enables findability, sharing and composition layers in the WoT. Objects can be discovered using already available search engines in addition to distributed discovery and lookup infrastructures. As with a user in a SNS that shares a Web resource, such as a webpage, the resources of a WoT-enabled thing can be shared. Describing resources using the same framework enables their inputs and outputs to be understood and composed easily. Hence, an important contribution of the WoT to the IoT is that it enables things to be accessed and understood anywhere using Web technologies. Once things are rendered homogeneously accessible, other valuable features can be developed. Nevertheless, the mechanism enabled by the WoT in terms of findability, at its current state, might need to integrate (or be integrated with) other approaches [38,43].

The SIoT promotes a scalable and flexible network structure between things [12,15]. The basic idea is that it enables each device to be part of a Social Network (SN) to search for required services or things (like friends in SNSs) and build reliable social relationships between them. The SIoT allows an autonomous and scalable search of services, as things search for the services they need incrementally, starting with themselves as the source node and then asking their friends for the services, the friends of their friends, and so on, biased by the trust assigned to each device. The scalability is possible thanks to the leverage of the small world phenomenon [39]. Furthermore, the SIoT gives “the IoT a structure that can be shaped as required to guarantee network navigability, so as that object and service discovery is effectively performed and scalability is guaranteed” [12]. Socially enabled things find eventually the services they need since there will be, eventually, a path between every two nodes [44].

## 3. Related Work

This work is a continuation of previous work [8], were we coined the term sioe to refer to the synergy between DSM and SIoT. On the one hand, a survey about SG scalability [45] reports that in terms of communication, the transition from a centralized architecture to a distributed one is necessary for a SG. Also, it reports that machine-to-machine communications (e.g., expressed in the SIoT paradigm), can open new opportunities and reduce communication costs. On the other hand, the main purpose of SIoT [12,15] is to provide the IoT with a social network structure that can be shaped as required and to provide better search efficiency. The SIoT also aims to provide autonomy and scalability to the search process.

To relate the contributions of this work with our previous work [8], we outline their respective contributions. We also outline our future work.

In [8], we:

- Explored how the SG can harness the SIoT technology to improve DSM services.
- Analyzed which technologies are involved when combining DSM and SIoT in a SG.
- Investigated a heuristic to elect members of a PCG via their energy profiles. On first attempts, the heuristic created empty clusters. Next, we developed a cluster-by-compatibility heuristic, summarized in Section 4.2, that addressed the problem of empty clusters. However, we did not evaluate if the PCGs created with that heuristic were able to achieve a global reduction in PAR.

In this work, we:

- Evaluate if the PCGs created by the cluster-by-compatibility heuristic can achieve a global reduction in PAR with two residential datasets.
- Perform the evaluation modifying an existing DSM algorithm. We modify the algorithm to be partially distributed.
- Observe that while we can achieve a global reduction in PAR, the optimal reduction (that occurs in a centralized algorithm) is hardly achieved.

Future work will aim at analyzing the composition of the PCGs that perform best in terms of reducing PAR. The analysis will allow us to direct our efforts to modify the heuristic and obtain a better composition of PCGs. The analysis will also allow us to develop dynamic clustering protocols to reconfigure the composition of PCGs.

The purpose of next sections is to position our work in relation to DSM scalability and SIoT communities.

### 3.1. Scalability in Demand Side Management

The authors in [10] present a partially distributed DSM algorithm which helps to aggregate underlying energy customers’ power and energy constraints and operating preferences. In contrast to alternative centralized methods, their approach requires less computational time to obtain decisions and scales well with an increase in network size. Also, they avoid a fully distributed approach as distributed approaches require several iterations to exchange locally optimized values. The authors propose to create MG that aggregate underlying users’ physical devices. After they obtain aggregated decisions for a model, these values are distributed among individual energy customers.

The consideration of MGs as the infrastructure to group customers has both advantages and disadvantages. On the one hand, it allows groups of customers in the same MG to detach from the main grid if necessary. For example, when a blackout occurs, customers in a detached MG can sustain their energy demands using the energy they generate through RES. On the other hand, and considering global energy management, it might be the case that a more optimal demand management could be achieved if customers were attached to another group (instead of the fixed one in a MG).

The authors in [11] also consider the communication/computation overhead in fully distributed DSM strategies. They apply a load-shifting DSM strategy while reducing costs and customer dissatisfaction. The authors propose groups of customers (a customer and its neighbors) to coordinate and estimate the baseline price in real time. Based on the estimated price and average energy consumption of the system, customers schedule their appliances to reduce costs and dissatisfaction level. On the supply side, the utility determines the exact price parameters based on customers’ consumption behavior and to make profit on the wholesale market.

Their approach does not need a concrete physical model for the electricity grid and, instead, harnesses the rapid-evolving communication network. However, they define the neighborhood of a customer as those customers connected by a local area network. In the sioe [8], we do not constrain the network to be local, only that at least one customer (more if the communication channel requires redundancy) is connected to the utility.

Some similarities can be drawn between [10,11] and our approach. We attribute reduction in communication/computation costs in the groups of customers and their local communication; these groups usually exchange aggregate information with the utility, although there might be situations that require one-to-one communication with the utility.

### 3.2. Social Internet of Things Communities

One of the fields of interest in SIoT is the creation of communities of objects to improve search efficiency; because objects in the same community share similar interests, the service searched for is more likely to be in the vicinity of the object that performs the search. We relate the creation of SIoT communities with the creation of PCGs. In the context of a PCG —and more specifically, in our work—the service searched for is a prosumer whose energy-consumption behavior is more likely to be compatible with the prosumer that performs the search; actually, is not the prosumer that searches, but the devices they own.

To create those communities, we need to investigate methods to group members with similar interests. As explained, in this work we shift the notion of similarity to the notion of compatibility. To provide with some background on community search, we highlight two of the latest works about this topic.

The authors in [46] propose algorithms to discover services among SIoT communities. They propose two types of ideas. First, they propose an algorithm to detect communities among established SIoT networks; and second, they propose algorithms to perform efficient service discovery among SIoT communities. On the one hand, they use three types of measurements to detect communities: preference similarity, location similarity, and social similarity. On the other hand, they address intra-community and inter-community search. If the search is within the local community, the device sends the query to its most immediate neighbors. If the search is outside the local community (meaning that the search does not have similarity with the interests of the local community), the device sends the query to the coordinators of other communities.

The authors in [47] propose a model to create dynamic communities of objects with similar interests. They explicitly consider the emergence of new objects in the network. When a new object emerges, surrounding objects become aware of it and vice versa, and the object checks if there is any community in the vicinity (within communication range) with similar interests. If not, the object tries to find other objects with similar interests. However, they can leave the community if there is a change on interests of the user (that owns the social devices), or based on social interaction rules, such as a limit on the number of communities.

To the best of our knowledge, there are no studies on how to create dynamic communities of PCGs in the context of SIoT. Therefore, this work, which analyses the implications of a heuristic in terms of DSM and PCG, is a necessary step towards the realization of this convergence.

## 4. Model

In this section, we describe the load model we use. The load model is not new and appears, more or less complete, on the works reviewed in Section 2. Furthermore, the model was presented in our previous work [8] were we only needed to model fixed loads. We update the model adding flexible loads. Fixed loads are loads that cannot be shifted in time, flexible loads (or time-shiftable loads) are those that can be shifted in time. For the sake of completeness, we describe the model in this section.

### 4.1. Load Model

We describe user and load characteristics as in [8]. Let U be the set of users and T the set of time slots, where U=|U| and T=|T|. (The letter in the left-hand side of = is the cardinality of the set on the right-hand side.) For each u∈U, we define the power consumption vector as

(1) $$\vec{l_{u}}=\left[l_{u}^{1},\ldots ,l_{u}^{t},\ldots ,l_{u}^{T}\right]$$

where $l_{u}^{t}$ is the energy consumption of user *u* at time slot *t*. As the model allows for appliance granularity, the set of appliances that belong to a user *u* are represented by $A_{u}$ and $A_{u}=\left|A_{u}\right|$. Then, $\vec{l_{u}}$ expands to the matrix

(2) $$\left[\begin{array}{ccccc} lm_{u_{1}}^{1} & \cdots & lm_{u_{1}}^{t} & \cdots & lm_{u_{1}}^{T}\\ lm_{u_{a}}^{1} & \cdots & lm_{u_{a}}^{t} & \cdots & lm_{u_{a}}^{T}\\ \vdots & \; & \vdots & \; & \vdots \\ lm_{u_{A_{u}}}^{1} & \cdots & lm_{u_{A_{u}}}^{t} & \cdots & lm_{u_{A_{u}}}^{T} \end{array}\right]$$

where *a* is a specific appliance of user *u* (e.g., washing machine). The load scheduling vector for all users U is $\vec{L}=\left[L^{1},\ldots ,L^{t},\ldots ,L^{T}\right]$ where

$$L^{t}=\sum_{u=1}^{U}l_{u}^{t}$$

$L^{t}$ is the total load in time slot *t*.

Total load per user u∈U during *T* time slots is denoted by

(3) $$L_{u}=\sum_{t=1}^{T}l_{u}^{t}$$

The PAR is then denoted by

(4) $$par{\left(u\right)}_{u\in U}=\frac{\max_{t\in T}l_{u}^{t}}{\frac{1}{T}L_{u}}$$

and is defined in a similar manner by each appliance at a more granular level and by each cluster in a less granular level. In fact, intra-cluster consumption can be represented by a vector similar to the one presented in Equation (1) and then dis-aggregated as per consumer in a similar manner as in Equation (2).

As a model improvement from previous work [8] and adapting some of the models described in [18] we differentiate from fixed loads and time-shiftable loads. Fixed loads are denoted by the set F and time-shiftable loads are denoted by the set S; where F=|F| and S=|S|. Hence, the set of all loads L is the union of fixed and time-shiftable loads, L=F∪S. For each user *u*, the set of loads is represented as $L_{u}$ and $L_{u}=F_{u}\cup S_{u}$.

As in [18], we consider time-shiftable loads, which usually represent appliances such as washing machine or dish washer, to have sub-tasks. For example, the washing machine can be used two times a day; we consider the washing machine as a task, and using it two times a day as two sub-tasks. We define $S_{u_{a}}=\left\{S_{u_{a}}^{1},\ldots ,S_{u_{a}}^{k},\ldots ,S_{u_{a}}^{q}\right\}$ as the set of *q* sub-tasks for a given task *a* of user *u*. Each sub-task is scheduled in $T_{u_{a}}^{k}$ timeslots, where $T_{u_{a}}^{k}=\left\{t\right|t_{u_{a}}^{s}\leq t\leq t_{u_{a}}^{e},t^{s}\geq 1,t_{u_{a}}^{e}\leq T,t_{u_{a}}^{e}=t_{u_{a}}^{s}+|T_{u_{a}}^{k}\left|\right\}$, and $T_{u_{a}}=\left\{T_{u_{a}}^{1},\ldots ,T_{u_{a}}^{k},\ldots ,T_{u_{a}}^{q}\right\}$ is the set of timeslots for each sub-task. $|T_{u_{a}}^{k}|$ is the duration of the sub-task $S_{u_{a}}^{k}$. Also, $T_{u}$ is the set of timeslots of each task in $S_{u}$. We assume non-overlapping sub-tasks. For any task $S_{u_{a}}$:

(5) $$\forall \left(T_{u_{a}}^{k},T_{u_{a}}^{m}\right)\in T_{u_{a}};k\neq m:T_{u_{a}}^{k}\cap T_{u_{a}}^{m}=⌀$$

**Figure 1:**
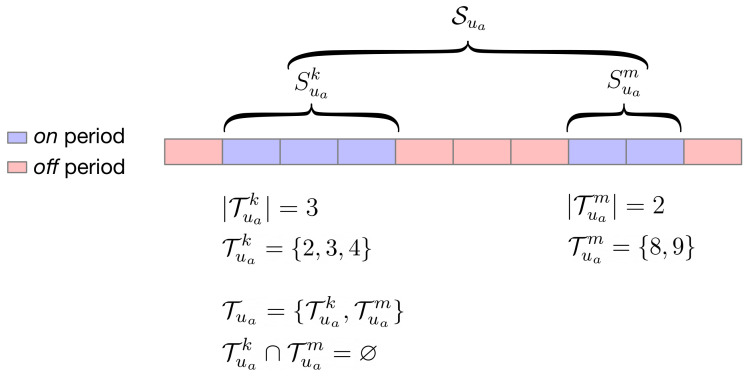
Task and sub-tasks.

Figure 1 depicts an example of the previous formulation for an appliance *a* of user *u*.

### 4.2. Clustering Model

The objective in [8] was to create clusters of prosumers as much compatible as possible. The set of load shapes is defined as L where $L=\{\vec{l_{u_{1}}},\ldots ,\vec{l_{u_{i}}},\ldots ,\vec{l_{u_{U}}}\}$. We define the set of clusters as C and its cardinality as K=|C|, where $C=\{C_{1},\ldots ,C_{k},\ldots ,C_{K}\}$ and $C_{k}\subset L$. Moreover, members of a cluster *k* cannot pertain to other clusters: $\forall \left(C_{k},C_{m}\right)\in C;k\neq m:C_{k}\cap C_{m}=⌀$. Given two or more prosumers x,y,z,… where x≠y≠z, we define their *compatibility* as how much they contribute in reducing the PAR of the sum of their power consumption vectors ($par(\vec{l_{x}}+\vec{l_{y}}+\vec{l_{z}}+\ldots )$. For each cluster *C*, it is desirable to maximize their compatibility, which means to minimize their PAR (so it gets as closer to 1, the lowest value). Given that each cluster is a subset of L, the compound PAR of all clusters is equal to par(L), Equation (6).

(6) $$\forall C_{k}\in C:par\left(⋃C_{k}\right)=par\left(C\right)=par\left(L\right)$$

In the same manner as $\vec{l_{u}}$ expands to $lm_{u}$ (Equation (2), a cluster C∈C can be collapsed by calculating the summation of all profile shapes $\vec{l_{u}}\in C$, that is the cluster profile shape. We will refer to the latter as

(7) $$\vec{C}=\sum_{u=1}^{\left|C\right|}\vec{l_{u}}$$

for each $\vec{C_{k}}\in C$. Also

(8) $$\vec{C}=\sum_{k=1}^{K}\vec{C_{k}}=\sum_{u=1}^{U}\vec{l_{u}}$$

The clustering algorithm we proposed on our previous work [8] is hierarchical and in a bottom-up approach. For the sake of simplicity, we treat the set L as a variable. An overview of the algorithm is as follows:

1. Choose one load $\vec{l_{u}}$ from the set of all loads L.
2. Find the best compatible load $\vec{l_{y}}$ in set $\left(L-\vec{l_{u}}\right)$. Create a cluster with $\left[\vec{l_{u}},\vec{l_{y}}\right]$.
3. The new set of loads L becomes $\left(L-\vec{l_{u}}-\vec{l_{y}}\right)$.
4. Repeat from (1) to (3) until L=⌀.
5. When L=⌀, add new clusters to L and repeat from (1) to (5) until the desired number of clusters *K* is obtained.

### 4.3. Load Rescheduling Algorithm

In our previous work we provided the complete description of the clustering algorithm; however, our attempts to apply a DSM algorithm to reduce the PAR were not successful. The reason was that we were applying a DSM algorithm to all the intermediate clusters without any coordination mechanism. The result was that the PAR was lower for each cluster, but not globally—which is one of the goals of the DSM algorithm. In this work, we develop a new algorithm. We base our rationale on the work done in [17] to explain the algorithm we develop. There, the authors present a straightforward rescheduling algorithm that evolves from centralized, to distributed, to partially distributed.

First, they consider a centralized algorithm to reschedule flexible loads and achieve a lower PAR. They consider all loads as input and reschedule them sequentially; once a load has been rescheduled, the new load to reschedule is positioned (in time) according to the previously rescheduled loads. The initial load shape is equal to the load shape of fixed loads. Their objective is not to reduce PAR but, instead, to reduce the mean square error between the rescheduled load shape and the ideal average load shape. The average load shape function sums all amplitudes of the users and divides the amplitude among *T* timeslots, obtaining a flat load shape.

They state that the rescheduling problem is NP-hard, each reschedule of a load conditions the position of the incoming ones. Therefore, they should try all possible combinations (i.e., all possible orders) of flexible loads to obtain the optimal solution. Consequently, they consider a greedy algorithm: they sort loads considering different load parameters before feeding them to the algorithm. (“A greedy algorithm always makes the choice that looks best at the moment, i.e., it makes a locally optimal choice in the hope that this choice will lead to a globally optimal solution” [48].) They argue that for some NP-hard problems, sorting objects in a certain manner can outperform other heuristics. Their results show a reduction in mean square error.

Secondly, they develop a distributed algorithm. They base the distributed algorithm on the centralized one, and apply the centralized algorithm to the loads of each user (individually). Users share the objective to minimize the error between their load shape and the average load shape of all users. To down-scale the amplitude for each user, they divide the average load shape by the total number of users. Therefore, users share the objective to minimize the error between their load shape and the average load shape per user.

However, the results do not show a reduction in mean square error. The rationale is that the shared objective does not contain information about where each user should move their flexible loads; each user positions their flexible loads without considering where other users positioned their loads. Therefore, due to the lack of coordination between users, the global mean square error is hardly reduced and, sometimes, even increased.

Thirdly, they modify the algorithm to be partially distributed. Because the distributed implementation lacks coordination between users, they introduce some coordination by the grid. They have each user perform a reschedule of its loads on top of the previously rescheduled loads of other users. Therefore, the user *u* reschedules their loads on top of the 1,…,u−1 loads of other users. When user *u* finishes the reschedule it sends the aggregated result to the grid and error towards average load to the grid. The results are similar to the ones obtained with the centralized algorithm. Nevertheless, the algorithm is sequential, and each user needs to wait for previous users to reschedule their loads.

The literature we explore in Section 2 shows a concern about scalability for centralized DSM algorithms. Continuing with the approach of recent literature, we develop a partially distributed algorithm based on the algorithms that [17] describes. The enhancement is that our model adds coordination by the grid and enables each PCG to reschedule their loads in parallel. The goal is to reduce PAR globally while allowing PCGs to perform local and autonomous reschedule of their loads.

The partially distributed implementation presented in [17] gets closer to one of the main objectives of the sioe: to make each PCG more autonomous and to allow distribution of computational resources. However, the algorithm they describe enforces a sequential and dependent rescheduling from other users, which does not promote autonomous PCGs. Furthermore, we relate central, distributed and partially distributed algorithms with three infrastructure configurations; cloud, fog and edge computing [49].

Cloud computing infrastructure matches a centralized algorithm; prosumers’ information is sent to the cloud and resulting, per prosumer information is returned. On the distributed algorithm, each prosumer performs the rescheduling of their flexible loads. Local, individual rescheduling is best described in a fog/edge IoT infrastructure; there, an aggregator owned by the user runs the algorithm. The partially distributed algorithm, as described in [17], off-loads the cloud by requesting each user to run the algorithm. The process is sequential, involving both cloud and fog/edge nodes at each iteration.

**Figure 2:**
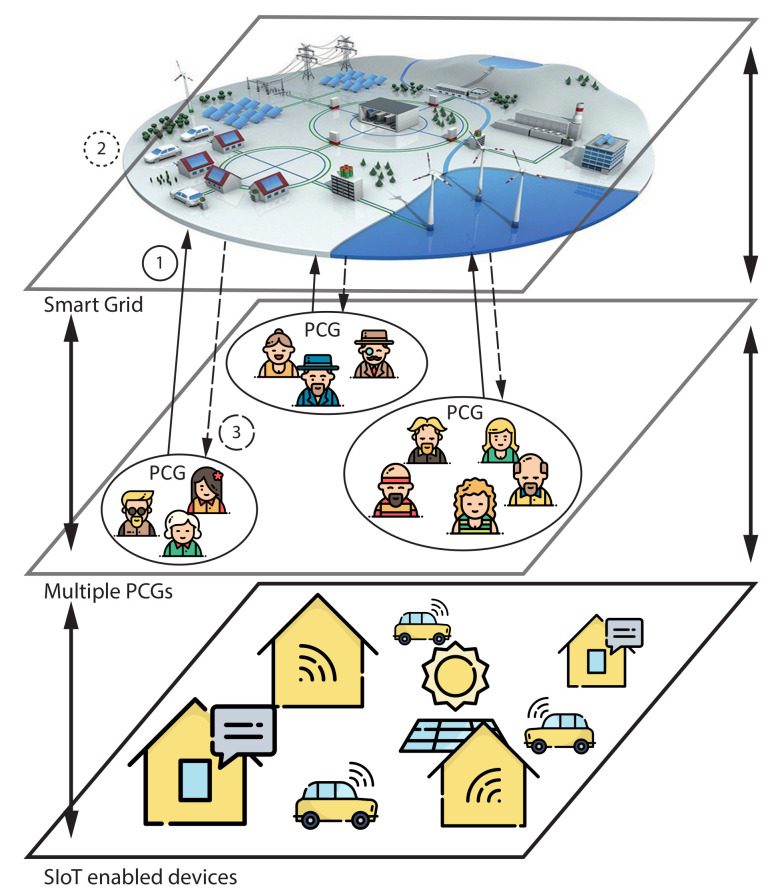
Multiple PCG structure with coordination. Numbers in circles indicate where each step is performed. Circle shapes for each step coincide with line shapes of the arrows. The arrows indicate the direction of the flow of information. The second step has no arrow. Each PCG sends information about local load profiles to a central node or coordinator (1). The coordinator assigns weight to when prosumers in a PCG should reschedule their loads (2). Weight information is sent back to each PCG, and they perform a local rescheduling of their loads (3).

On the contrary, our approach (explained in the following sections) considers a fog infrastructure where nodes are associated with PCGs in a one-to-one relationship. Fog nodes can run a local rescheduling algorithm in parallel based on a coordination mechanism executed by a central resource; potentially embodied by a cloud infrastructure (Smart Grid or cloud layer in Figure 2).

Based on the algorithms described in [17], we propose a novel partially distributed algorithm that uses a central node as the coordination mechanism. However, it allows each PCG to autonomously schedule loads of their users. We propose a system of weights where each user is assigned a weight or likelihood at specific timeslots. The weight system aims to influence the load rescheduling of each user, so they work together to improve the total PAR without communicating between them. We split the algorithm into two phases. Although we use the same algorithm for both phases, what differentiates each phase is the treatment of input data and output results. The first phase assigns weights to certain timeslots for users in a PCG; the second phase reschedules time-shiftable loads of each user, where weights influence the position shifted to.

The interaction between the coordinator and PCGs is modeled in three steps. The reader can refer to Figure 2 along the explanation; each step corresponds to an encircled number. During the first step, each PCG sends information about its load profile (aggregated load profiles of each user in the PCG) to a coordinator. The second step uses this information to assign weights for each PCG; weights are assigned to certain timeslots for each PCG. Finally, the third step refers to PCGs performing local rescheduling to decrease global PAR. They do so by skewing their load curve according to assigned weights.

#### 4.3.1. PCG Representation

Once PCGs are created, each one of them sends information about the loads of their users to the coordinator. (Figure 2 depicts this step with an encircled number 1.) The information they send is:

- the cluster profile shape $\vec{C}$ of fixed loads $F_{C}$ within the cluster (the aggregate of $F_{C}$) and,
- the total energy consumed by the flexible loads of the cluster and minimum and maximum time-span in which all flexible loads in the cluster $S_{C}$ (of cluster *C*) can be rescheduled. The minimum time-span $D^{min}$ occurs when all sub-tasks of users in the cluster are scheduled at the same time period, and the maximum time-span $D^{max}$ occurs when all subtasks of users in the cluster are scheduled one after the other (or, if the total duration exceeds *T*, scheduled across all timeslots T). We will refer to this information as the *PCG representation information*, which is represented as a flexible load.

#### 4.3.2. Weight Allocation

Then, during step two, the coordinator uses Algorithm 1 to assign weights for each PCG. (Figure 2 depicts this step with an encircled number 2). Algorithm 1 is based on the one described in [17]. Please note that for all algorithms described in this article, operations without an equals sign (e.g., +) are immutable, meaning that in “cAux = cAux + *s*”, the + operation performs a copy of *cAux* and adds *s*, while the = operation assigns the copy to *cAux*. Because we represent clusters of loads as sets of loads, it is worth noting that operations such as +s to a given set that already contains *s*, replace the old instance of *s* instead of doing nothing. Equality of loads (and thus set operations) is performed based on their unique identifiers instead of other structural properties. To emphasize that $t_{u_{a}}^{s}$ and $t_{u_{a}}^{e}$ are properties of a sub-task, namely *s*, we use the dot notation (e.g., s.$t_{u_{a}}^{s}$ reads: the start time of sub-task *s* of appliance *a* of user *u*).

The algorithm used in step two (Algorithm 1) takes a set of aggregated loads: a set of fixed loads and a set of flexible loads. Then, it is instructed to find the best starting time $t_{u_{a}}^{s}$ for each *PCG representation information*, which is transformed into a flexible load. To find the best starting time, we want to schedule each PCG to minimize peaks, i.e., we want to position flexible loads where $max(\vec{C})$ is minimized. The input parameters for Algorithm 1 are:

- F: the set of all fixed loads
- S: the set of PCG representations. One cluster is represented as one PCG representation information.
- t(C,sr): a function that transforms a PCG representation information into a flexible load. Algorithm 2 describes the heuristic.

**Algorithm 1:** PCG weight assignment algorithm.


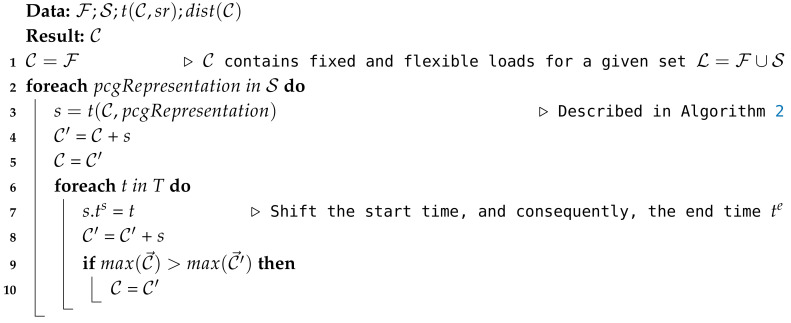



For Algorithm 2, *sr* is the *PCG representation information*. To emphasize that $D^{min}$ and $D^{max}$ are properties of *sr*, we use the dot notation (e.g., sr.$D^{min}$ reads: the minimum duration of the *PCG representation information*). sr.$L_{C}$ is the total energy consumed by the PCG, and the function averageFlexibleLoadOf receives two parameters, a duration and an amount of energy, and creates a load $\vec{l_{u}}=\left[l_{u}^{1},\ldots ,l_{u}^{t},\ldots ,l_{u}^{T}\right]$ (see Equation (1)) where *T* is equal to the duration parameter and each component $l_{u}^{t}$ is equal to the amount of energy parameter divided by *T*. The reason for Algorithm 2 to consider both sr.$D^{min}$ and sr.$D^{max}$ is to consider edge cases. If sr.$D^{min}$ is used, the resulting weight indicates that all flexible loads of a given cluster should be scheduled during the same time period, as “one on top of the other”. If there is the chance that rescheduling the resulting flexible load (produced by averageFlexibleLoadOf), even at the lowest peak of the loads that C contains, the peak increases, then sr.$D^{max}$ is used instead. The resulting weight using sr.$D^{max}$ indicates that all flexible loads of a given cluster are spread so there is the less possible overlapping between them and the possible peaks are lowest. Also, observe that the transformation performed in Line 3 of Algorithm 1 considers transformation decisions (i.e., the ones made in Algorithm 2).

**Algorithm 2:** Flexible load representation transformation function.

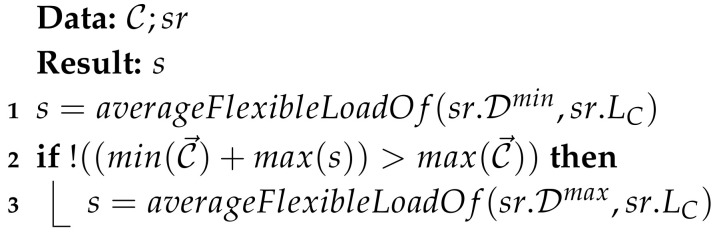



Once Algorithm 1 is applied during step two, C contains a set of flexible loads that represent each PCG. Then, these representations are converted into weights for each PCG. The weight set for each PCG $W_{C}$ is equal to the timeslots where *s* is scheduled. For example, Tseq is the sequence of available time slots (1,2,3,4); $\vec{F}=\left[1,0,0,1\right]$ is the load vector of F. The best possible position to schedule a flexible load $\vec{s}=\left[1,1\right]$ is at start position 2. Then, $\vec{F}+\vec{s}$ is equal to $\left[1^{1},0^{2},0^{3},1^{4}\right]+\left[1^{2},1^{3}\right]=\left[1^{1},1^{2},1^{3},1^{4}\right]$, where each value corresponds to the energy consumption at the time slot indicated by the super index. Then, $W_{C}=\left[2,3\right]$.

#### 4.3.3. PCG Rescheduling

Weights for each PCG are used during step three using Algorithm 3. (Figure 2 depicts this step with an encircled number 3.) Weight information $W_{C}$ is sent back from the coordinator to each PCG. Therefore, each PCG is able to reschedule the loads of its members both pursuing a reduction in local PAR and a reduction in global PAR using the weights assigned by the coordinator. Each PCG can use Algorithm 3 to reschedule their flexible loads. The flexible loads of a PCG are the flexible loads of its users, and each flexible load or task might contain several sub-tasks. Sub-tasks that pertain to the same task cannot overlap (for example, if a prosumer turns on the washing machine two times, those two sub-tasks cannot start at the same time, nor can overlap). Input parameters for Algorithm 3 at step three are:

- F: the set of all fixed loads for a given cluster ($F_{C}$).
- S: the set of all flexible loads (as sub-tasks) for a given cluster ($S_{C}$). For the sake of simplicity, we have omitted the constraint of non-overlapping sub-tasks expressed in Equation (5), but note that this constraint applies during this algorithm.
- W: the set of weights for a given cluster ($W_{C}$).
- *r*: the reference average. The concept of $\hat{R}$ for each cluster. If $\hat{R}$ is the average ideal load, $r=\frac{\hat{R}}{K}$ is the average ideal load per cluster.
- dist(C,W,r,s): A function that weights its result on the basis of weights W for a given cluster. The definition is given in Algorithm 4.

**Algorithm 3:** Rescheduler algorithm.

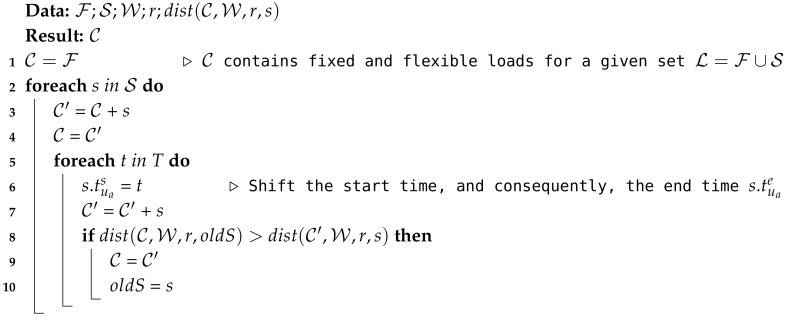



On step three, we change the distributed algorithm that [17] describes to take the additional inputs of weights and reduce the distance to the ideal average load. In Algorithm 4, we decrease the error by computing the overlap ratio between the individual load (a load of a time-shiftable appliance) and the positions with weight. Then, if *e* is equal to the mean square error and overlapRatio (that ranges from 0.0 to 1.0) the overlap between the position of the time-shiftable load and the weighted positions, the final error fe is fe=e+e*(1−overlapRatio). The algorithm finds the best position of the time-shiftable load when the distance is minimum compared with all other possible positions.

The mechanics we proposed include three steps that interact with three types of entities: the grid, PCGs and SIoT-enabled devices. The reader can follow the next description in Figure 2. First, each PCG sends information about the aggregated load shape of its prosumers to the grid; secondly, the grid uses the algorithm to assign weights to each PCG by rescheduling aggregated load shapes; and, thirdly, each PCG reschedules the load of its prosumers according to those weights. Therefore, the grid uses the algorithm to weight the positions where individual loads should be moved to, and the load curve of each PCG is skewed to obtain a flat load curve globally.

**Algorithm 4:** Weighted distance. **Data**: C;W,r,s
 **Result**: weightedD
**1** d = $\sum_{t=s.t_{u_{a}}^{s}}^{s.t_{u_{a}}^{e}}{\left(l_{c}^{t}-r\right)}^{2}$;
**2** overlapRatio = $\frac{|W\cap s.T_{u_{a}}^{k})|}{s.|T_{u_{a}}^{k}|}$
**3**weightedD=d+d∗(1−overlapRatio);

## 5. Experimentation

To test the rescheduling method proposed in Section 4.3, we use two datasets, one with synthetic data and the other with real data. We expect PCGs to coordinate and reduce global PAR.

For those datasets, we need to split the flexible loads in durations less than *T*, otherwise, we cannot advance or delay the start time of flexible loads (the start position in T). To do so, we assume that flexible appliances are *off* when their power consumption level is equal to the lowest value found in ${\vec{l}}_{u}$ and they are *on* when their power consumption level is different than the lowest value. Consecutive non-lowest consumption defines a duration of an *on* period, and thus the duration of a sub-task. Therefore, we split the initial time-shiftable load of duration *T* into smaller time-shiftable loads with lower duration ($|T_{u_{a}}^{k}|<T$); thus, they can move forward or backwards in the line defined by *T* consecutive numbers.

We have performed experiments taking samples from 50% to 100% of the total dataset at 10% steps. The sampling procedure from 50% to 100% shuffles the entire dataset and grabs the desired number of samples. Then the algorithm is applied for K=[1,6]. The procedure is repeated 200 times per each sample size and *k*. Also, each time we repeat the procedure (each of 200 times) the sample is the same. We differentiate between two procedures, the clustering procedure and the rescheduling procedure. In the text and figures that follow we refer to the clustering procedure as “s1” and to the rescheduling procedure as “s2”.

In running the experiments, we first cluster households using the hierarchical algorithm described in [8], then we run the rescheduler described in this work. The rescheduling optimization problem is NP-hard; consequently, we sort loads before running the rescheduler. For k=1 there is no coordination possible, and the loads are sorted by: (i) total energy and, (ii) $t_{u_{a}}^{s}$ , in both increasing and decreasing order. Hence, the algorithm is run 4 times per iteration on sample size, and the solution with the best PAR is taken.

For k=1 the algorithm we use is the centralized one, without any coordination step; this is because there is only one cluster of all users in the sample. Therefore, we can consider the case for k=1 as the reference case, and the results we obtain will serve to compare the results for combinations of k≠1 and sample size. We expect to see a trade-off between the reduction in PAR and the potential parallelization of the rescheduling procedure, which each PCG can perform autonomously.

### 5.1. Synthetic Dataset

We use the synthetic load generator developed in [50] to test the algorithm. In [50] synthetic residential loads are generated at a resolution of a 1-min interval for 24 h. The model is based on a correlation and combination of patterns of active occupancy and daily activity profiles. The model is also validated using comprehensive validation of statistical characteristics with a dataset containing real measurements. The synthetic generation can be configured with occupancy level, the month of the year and week or weekend day. The output of the simulation comprises load profiles for a set of 33 electrical appliances and a set of light bulbs.

We simulate the appliances of 200 users (U=200) and the appliance time resolution is 30 min. We can achieve that by adding amplitudes at batch interval of 30 min; hence, T=48. From the electrical appliances, we have fixed the number of flexible loads to 4 (S=4), e.g.,: dishwasher, tumble dryer, washing machine and washer dryer. The remaining 29 (F=29) appliances are set to fixed loads. Light bulbs are also represented as fixed loads.

**Figure 3:**
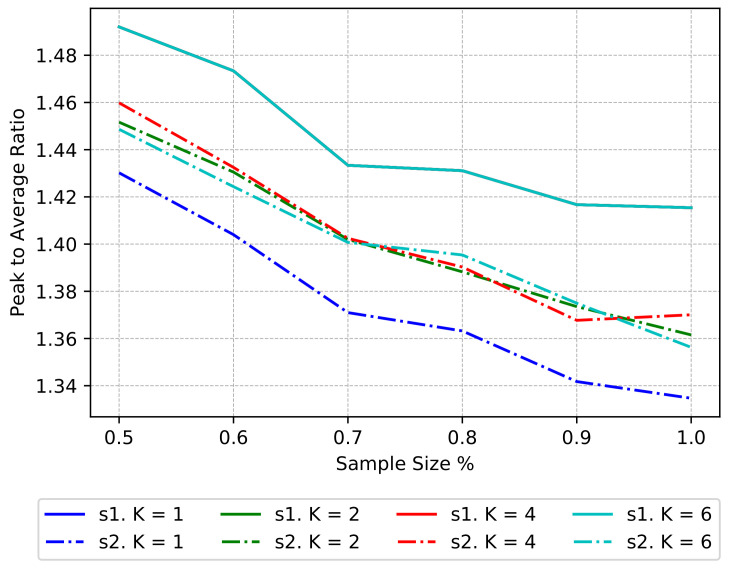
Mean PAR for “s1” (only clustering, before rescheduling) and “s2” (after scheduling) and K={1,2,4,6} with synthetic dataset.

Figure 3 shows the average reduction in global PAR for combinations of *k* and sample size. We can observe that for “s1” the lines of each *k* are totally overlapped; this is because each time we repeat the process the sample is the same and the PAR of the entire sample, regardless of the number of clusters, is the same. Also, we see that as the number of elements per sample grows, the PAR diminishes. We depict the characteristics of the data in the lines “s1”. The lines “s2” show the result of the clustering procedure in combination with the rescheduling procedure. As already mentioned, the reference case is when k=1, which shows the maximal reduction in PAR that can achieve the centralized algorithm. For k≠1 there is a reduction in PAR, and it is interesting to see that regardless of the sample size, the mean reduction in PAR is the same for different *k* (i.e., “s2” lines overlap).

**Figure 4:**
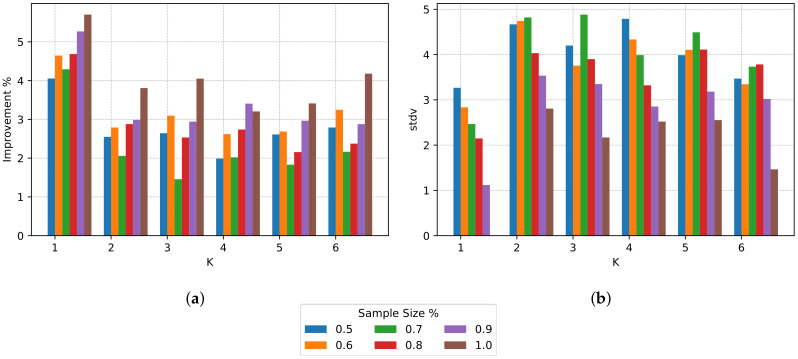
Improvements mean and associated standard deviation for different *K* and sample size for the synthetic dataset. (**a**) Mean of improvements; (**b**) Standard deviation of improvements.

We take a closer look at the improvement ratio and associated standard deviation in Figure 4a,b respectively. The improvement is maximum when k=1 and sample size = 1.0; also, the standard deviation is 0, showing that the centralized algorithm is deterministic. For k=1 and sample sizes different than 1.0, the standard deviation increases because the samples do not contain the same elements in each iteration. We expected an increase in improvement for bigger sample sizes and a decrease in improvement for bigger *k*s; nevertheless, there is not a clear impact between an increase in the number of clusters and the sample size, and the mean improvement.

The reader can refer to Table 1 to inspect the data present in Figure 3 and Figure 4. Table 1 shows, for each *k* and sample size (s. size in the table) ranging from 0.5 (50%) to 1.0 (100%), four measures. Rows “s1. mean” and “s2. mean” correspond to the information shown in Figure 3, and describe the average reduction in global PAR for combinations of *k* and sample size. Rows “impr. mean” and “impr. stdv” correspond to the information shown in Figure 4a,b respectively, and describe the improvement ratio and associated standard deviation.

**Figure 5:**
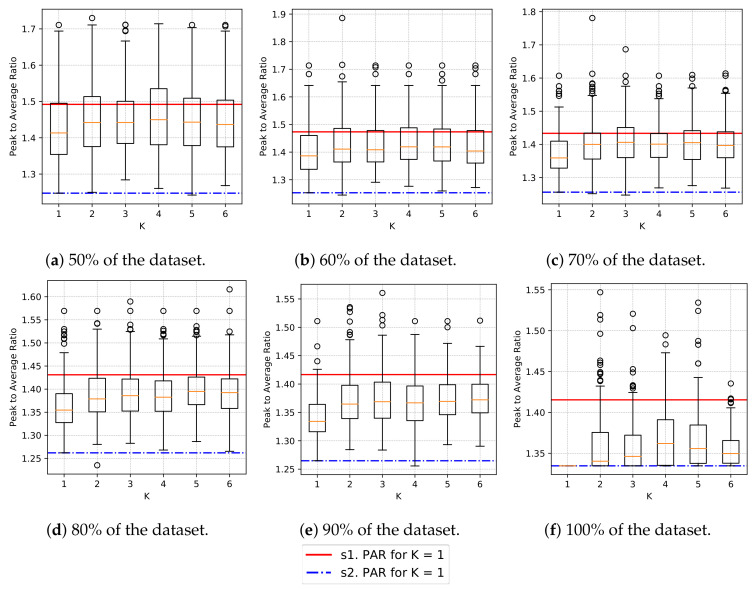
For the synthetic dataset, distribution of the achieved PAR after the rescheduling algorithms is applied. Red straight line describes the minimum PAR for *K* = 1 given the sample size at the stage “s1”. Blue dash-dot line describes the minimum PAR for *K* = 1 given the sample size at the stage “s2”.

As expected, we can see in Figure 4b that the standard deviation decreases as the sample size grows, because the probability of taking the same elements in other iterations increases. One can also observe this tendency in Figure 5, as the sample size grows, the results after load rescheduling fall below the mean PAR of unscheduled samples. Furthermore, the quartile distribution shows that maximum improvement occurs when sample size is 100% and minimal standard deviation also occurs when sample size is 100%.

**Figure 6:**
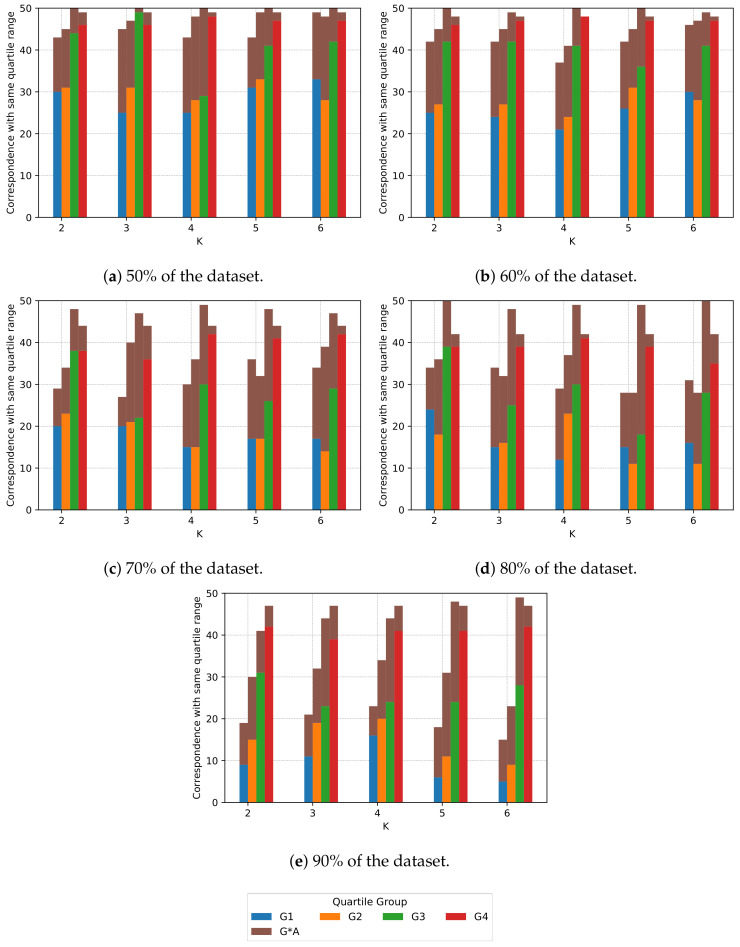
For the synthetic dataset, correspondence with inter-quartile range. “G1” reads inter-quartile group 1, “G2”, inter-quartile group 2, and so on. “G*A” accounts for the number of iterations we the result for *k* ≠ 1 has fallen in the range of a higher quartile.

We can observe the relation between the increase in the number of clusters and the PAR after rescheduling the loads within the same iteration in Figure 6, i.e., for each sample size and iteration: first, we consider within which inter-quartile range is the achieved PAR for k=1; and, secondly, for each k≠1 we look at how many iterations still are within the same inter-quartile group (non-brown bars) and the consecutive group (brown bars).

We observe in Figure 6 that for small sample sizes, the inter-quartile group is more likely the same for different *k*s. However, as the sample size increases (from 50% to 90%), the likelihood of being in the same inter-quartile group decreases. Moreover, the quantity of results that are not in the immediate higher inter-quartile group (for example, that should remain in G2 but move to G4) also increases.

If we combine the information shown in Figure 5 and Figure 6 regarding the increase in sample size, we observe that while there is a slight “movement” of the distribution towards the minimum for “s2” (blue line) (and thus, the inter-quartile groups are closer to the best solution), the likelihood that the inter-quartile group is the immediate superior increases, i.e., brown bars in Figure 6 increase in height as the sample size increases. Furthermore, the height without bars until 50 samples indicates that the result for that sample “moves” even further away from the next consecutive inter-quartile group (e.g., from G2 to G4).

The reader can refer to Table 2 to inspect the data present in Figure 6. Table 2 shows, for each sample size (s. size in the table) and for each *k*, the number of iterations when k≠1 that are in the same inter-quartile range (G1, G2, G3 or G4) as the inter-quartile ranges for k=1. The table also shows the number of iterations that fall in the next consecutive group (G*A).

As we state in Section 1, our objective is not to improve on DSM algorithms, but to validate that we can achieve cluster configurations that achieve the lowest PAR if prosumers are grouped by compatibility. Furthermore, if we group them by compatibility, they will have to perform less changes in their behavior to achieve a global reduction in PAR.

From the results we conclude that we can obtain cluster configurations that achieve the minimal PAR, and that more of 50% of the times (worst case in Figure 6e) optimal or better-than-initial configurations are maintained across different values of *k*.

### 5.2. Real Dataset

To validate those results with real data, we use part of the large dataset provided by Dataport [51]. We select households which have a smart meter installed with individual circuits for each appliance. 189 enrolled households (U=189) in 1 January 2015 had a smart meter (eGauge device) with individual circuits for each appliance. eGauge readings are per hour, meaning that each record contains power readings for up to 12 circuits (appliances) during 24 timeslots (T=24). From those 12 appliances ($A_{u}=12$), we assume that 5 of them are flexible (S=5) and the others fixed loads (F=7). According to the names that identify each appliance in the Dataport database, we assume that “clotheswasher1”, “clotheswasher_dryg1”, “drye1”, “dryg1” and “dishwasher1” are flexible appliances.

The results are similar to the ones obtained using the synthetic dataset. We highlight some similarities and differences.

**Figure 7:**
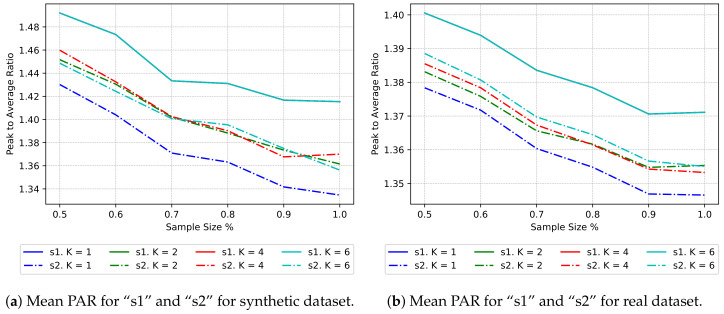
Mean PAR for “s1” and “s2” and K={1,2,4,6} Comparison between synthetic and real datasets.

**Figure 8:**
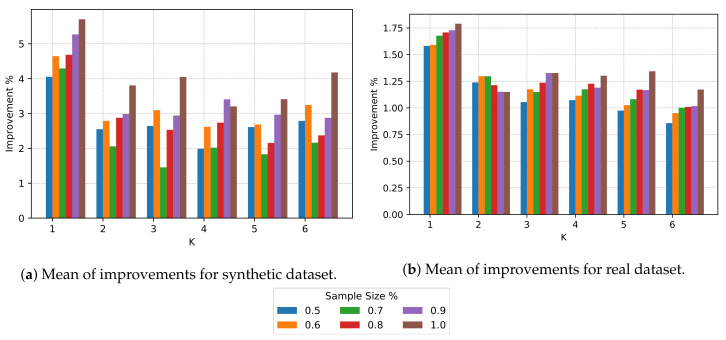
Improvements mean. Comparison between synthetic and real datasets.

As shown in Figure 7, the rescheduler algorithm achieves a reduction in PAR on average. However, the sample size has a lesser impact on the average reduction in global PAR than with the synthetic dataset (see Figure 8). We argue that this is due the differences in the datasets and the timeslot resolution; T=24 for the real dataset, and T=48 for the synthetic dataset. For each sample size, we also observe that there is a slight reduction in improvement as *k* grows, which indicates that the number of clusters has an impact on the improvement ratio (Figure 8b).

The quartile group distribution is similar to the one described for the synthetic dataset; as the sample size grows, inter-quartile groups are closer to the reference value for “s2”, as in Figure 5. Furthermore, the correspondence between inter-quartile ranges is similar for both datasets; as the sample size grows, the inter-quartile group is less likely to be the same for different *k*’s, as in Figure 6.

Finally, Table 3 and Table 4 are analogous to Table 1 and Table 2, but using the real dataset.

### 5.3. Discussion

Both results, for synthetic and real datasets, show that the load rescheduling process we propose can achieve a reduction in PAR. The algorithm also achieves a better-than-initial PAR in most cases, and the correspondence between inter-quartile groups for different values of *K* is high; however, as the sample size increases, the correspondence decreases, as seen in Figure 6. Furthermore, we observe that in most cases, our algorithm can achieve clusters that reach the minimal PAR, we will investigate which are the properties of the clusters that achieve the lowest PAR. Because we obtain similar results for both datasets, we expect that our three-step algorithm will be able to obtain similar results for other residential datasets.

Also, these results show that after a single and low resource-consuming coordination (because prosumers in each PCG are aggregated when the coordination process takes place), each PCG can reschedule their loads independently, regardless of the other PCGs, i.e., in parallel with other PCGs, which allows distributing computation resources. Indeed, the rescheduling algorithm is implemented (prototyped) in such a manner that after coordination, a dedicated thread performs the rescheduling for each cluster.

As highlighted in Section 3, there is a growing concern about the amount of computation and network resources necessary to carry out centralized and distributed DSM algorithms. Therefore, some DSM solutions, besides considering energy efficiency, consider computation and network usage. The approach we take in modeling the DSM algorithm considers both computation and network resources. We consider computation resources by distributing the tasks among several PCGs. Moreover, we consider network resources by only communicating information with a coordination entity on only one round-trip.

However, we know that there must be a trade-off between a centralized solution and a distributed one, as seen for the cases when k=1 (centralized) and k≠1 (distributed) in the experiments, where the centralized algorithm achieves a better reduction in PAR. Nonetheless, centralized solution does not allow parallelization of the computation, while a distributed one does (i.e., creating clusters that perform the rescheduling process autonomously). However, if we want to avoid communication overhead between the distributed clusters, we will not be able to achieve the same optimal as with a centralized manner.

Therefore, the rescheduling process might not only be performed in a centralized way, as in the cloud, but in a decentralized manner using fog computing at the edge and near each PCG in a network-sustainable manner, if it is the case that each PCG is formed using geographical constraints. Fog nodes are IoT aggregators which run the local rescheduling algorithm (step 3 in Figure 2). Edge nodes are sensors and actuators in the IoT: electrical appliances sending power consumption data and even remotely actionable (e.g., on/off) by the prosumer or utility.

Moreover, and as observed in [17], if clusters (users in their paper) do not share any context information about other clusters or about a common goal—about where they should aim as a group of clusters—a global reduction in PAR might only be achieved randomly; each cluster can achieve a relative optimal solution, but not the global optima. For example, with the presented algorithms, a utility could articulate and coordinate multiple geographical areas towards the reduction of PAR and reducing their energy production costs.

## 6. Conclusions and Further Work

This work is a continuation of previous work [8], which coins the term sioe to refer to the application of SIoT [12] to SGs and, more concretely, to DSM. The goal of [8] and this work is to provide an overlay social network that facilitates virtual connection between smart devices, prosumers and PCGs. This virtual connection could facilitate the scalability, decentralization and distribution of SG services, such as DSM.

We develop a load rescheduling algorithm that allows us to analyze the implications of a heuristic to cluster prosumers by compatibility. The load rescheduling algorithm we develop comprehends several PCGs with a common goal and an entity to coordinate those groups. The goal to reduce PAR globally demands action from each PCG towards that goal. Moreover, this separation by PCG allows each one of them to execute the rescheduling process semi-autonomously, with only the guidelines that the entity that coordinates them provides to achieve the common goal.

We test the algorithm with multiple samples of synthetic and real datasets concerning residential electricity demand. For each sample, we analyze the if the PAR for an increasing number of clusters is within the same range as if there were only one cluster (the near-optimal solution, since the problem of load rescheduling is NP-hard). The results for both synthetic and real datasets are similar, and show that we can achieve an optimal cluster configuration using the clustering-by-compatibility heuristic.

These results will allow us to keep our investigations on the differences on the elements of the clusters that achieve the same PAR as the best that can be achieved (with the centralized algorithm) and the clusters that move away from the optimal PAR.

## Figures and Tables

**Table 1:** Synthetic dataset. Improvements.

	s. Size	0.5	0.6	0.7	0.8	0.9	1.0
k	Measure						
1	impr. mean (%)	4.05	4.65	4.29	4.68	5.27	5.70
	impr. stdv	3.27	2.83	2.47	2.15	1.12	0.00
	s1. mean	1.49	1.47	1.43	1.43	1.42	1.42
	s2. mean	1.43	1.40	1.37	1.36	1.34	1.33
2	impr. mean (%)	2.55	2.79	2.06	2.88	2.99	3.81
	impr. stdv	4.67	4.74	4.82	4.03	3.53	2.81
	s1. mean	1.49	1.47	1.43	1.43	1.42	1.42
	s2. mean	1.45	1.43	1.40	1.39	1.37	1.36
3	impr. mean (%)	2.64	3.10	1.46	2.53	2.94	4.05
	impr. stdv	4.20	3.75	4.88	3.90	3.35	2.17
	s1. mean	1.49	1.47	1.43	1.43	1.42	1.42
	s2. mean	1.45	1.43	1.41	1.39	1.37	1.36
4	impr. mean (%)	1.99	2.62	2.02	2.74	3.41	3.20
	impr. stdv	4.79	4.33	3.99	3.32	2.85	2.52
	s1. mean	1.49	1.47	1.43	1.43	1.42	1.42
	s2. mean	1.46	1.43	1.40	1.39	1.37	1.37
5	impr. mean (%)	2.61	2.69	1.83	2.16	2.97	3.41
	impr. stdv	3.99	4.10	4.49	4.11	3.18	2.55
	s1. mean	1.49	1.47	1.43	1.43	1.42	1.42
	s2. mean	1.45	1.43	1.41	1.40	1.37	1.37
6	impr. mean (%)	2.79	3.25	2.16	2.37	2.88	4.18
	impr. stdv	3.47	3.34	3.73	3.78	3.02	1.46
	s1. mean	1.49	1.47	1.43	1.43	1.42	1.42
	s2. mean	1.45	1.42	1.40	1.40	1.37	1.36

**Table 2:** Synthetic dataset. Inter-quartile groups movement.

	IG	G1	G1A	G2	G2A	G3	G3A	G4	G4A
s. size	k								
0.5	2	30	13	31	14	44	6	46	3
	3	25	20	31	16	49	1	46	3
	4	25	18	28	20	29	21	48	1
	5	31	12	33	16	41	9	47	2
	6	33	16	28	20	42	8	47	2
0.6	2	25	17	27	18	42	8	46	2
	3	24	18	27	18	42	7	47	1
	4	21	16	24	17	41	9	48	0
	5	26	16	31	14	36	14	47	1
	6	30	16	28	19	41	8	47	1
0.7	2	20	9	23	11	38	10	38	6
	3	20	7	21	19	22	25	36	8
	4	15	15	15	21	30	19	42	2
	5	17	19	17	15	26	22	41	3
	6	17	17	14	25	29	18	42	2
0.8	2	24	10	18	18	39	11	39	3
	3	15	19	16	16	25	23	39	3
	4	12	17	23	14	30	19	41	1
	5	15	13	11	17	18	31	39	3
	6	16	15	11	17	28	22	35	7
0.9	2	9	10	15	15	31	10	42	5
	3	11	10	19	13	23	21	39	8
	4	16	7	20	14	24	20	41	6
	5	6	12	11	20	24	24	41	6
	6	5	10	9	14	28	21	42	5

**Table 3:** Real dataset. Improvements.

	s. Size	0.5	0.6	0.7	0.8	0.9	1.0
k	Measure						
1	impr. mean (%)	1.72	1.70	1.66	1.73	1.75	1.79
	impr. stdv	0.62	0.48	0.39	0.33	0.22	0.00
	s1. mean	1.40	1.40	1.38	1.38	1.37	1.37
	s2. mean	1.38	1.37	1.36	1.35	1.35	1.35
2	impr. mean (%)	1.34	1.36	1.29	1.27	1.24	1.30
	impr. stdv	0.87	0.88	0.80	0.69	0.74	0.69
	s1. mean	1.40	1.40	1.38	1.38	1.37	1.37
	s2. mean	1.38	1.38	1.36	1.36	1.36	1.35
3	impr. mean (%)	1.18	1.28	1.32	1.30	1.34	1.38
	impr. stdv	0.91	0.76	0.61	0.71	0.62	0.61
	s1. mean	1.40	1.40	1.38	1.38	1.37	1.37
	s2. mean	1.39	1.38	1.36	1.36	1.36	1.35
4	impr. mean (%)	1.34	1.27	1.24	1.28	1.22	1.36
	impr. stdv	0.83	0.81	0.67	0.69	0.68	0.59
	s1. mean	1.40	1.40	1.38	1.38	1.37	1.37
	s2. mean	1.38	1.38	1.36	1.36	1.36	1.35
5	impr. mean (%)	1.17	1.25	1.23	1.35	1.27	1.37
	impr. stdv	0.83	0.69	0.63	0.64	0.58	0.50
	s1. mean	1.40	1.40	1.38	1.38	1.37	1.37
	s2. mean	1.39	1.38	1.36	1.36	1.36	1.35
6	impr. mean (%)	1.20	1.25	1.21	1.27	1.29	1.33
	impr. stdv	0.82	0.69	0.57	0.59	0.48	0.52
	s1. mean	1.40	1.40	1.38	1.38	1.37	1.37
	s2. mean	1.39	1.38	1.36	1.36	1.36	1.35

**Table 4:** Real dataset. Inter-quartile groups movement.

	IG	G1	G1A	G2	G2A	G3	G3A	G4	G4A
s. size	k								
0.5	2	42	6	43	7	43	6	48	2
	3	45	4	44	6	39	10	48	2
	4	45	4	43	7	47	3	49	1
	5	40	9	41	9	44	6	48	2
	6	41	8	42	8	44	6	48	2
0.6	2	44	3	44	5	40	9	46	3
	3	39	10	43	7	41	9	45	4
	4	42	6	38	12	44	6	48	1
	5	43	6	34	16	41	8	46	3
	6	46	3	41	9	38	12	45	4
0.7	2	40	7	39	8	38	12	49	1
	3	39	8	43	5	43	6	49	1
	4	37	11	40	10	39	10	49	1
	5	35	11	39	10	39	11	49	1
	6	38	10	38	12	38	11	49	1
0.8	2	36	11	33	8	36	14	48	2
	3	37	12	32	13	34	16	48	2
	4	40	10	37	10	38	12	47	3
	5	38	11	36	12	34	16	46	4
	6	41	9	33	15	30	20	46	4
0.9	2	31	7	32	9	34	16	42	8
	3	34	12	32	12	37	13	43	7
	4	26	17	28	15	32	17	42	8
	5	35	9	24	25	31	18	46	4
	6	31	17	24	20	26	24	45	5
